# Prognostic Factors for Return to Work, Sickness Benefits, and Transitions Between These States: A 4-year Follow-up After Work-Related Rehabilitation

**DOI:** 10.1007/s10926-013-9466-5

**Published:** 2013-08-09

**Authors:** Irene Øyeflaten, Stein Atle Lie, Camilla M. Ihlebæk, Hege R. Eriksen

**Affiliations:** 1 The National Centre for Occupational Rehabilitation, Haddlandsvegen 20, 3864 Rauland, Norway; 2 Department of Health Promotion and Development, University of Bergen, Bergen, Norway; 3 Uni Health, Uni Research, Bergen, Norway; 4 The Section for Public Health Science, ILP, University of Life Sciences, Aas, Norway

**Keywords:** Sick leave, Disability leave, Return to work, Rehabilitation—Vocational, Risk factors

## Abstract

*Purpose* The aim of this study was to examine if age, gender, medical diagnosis, occupation, and previous sick leave predicted different probabilities for being at work and for registered sickness benefits, and differences in the transitions between any of these states, for individuals that had participated in an interdisciplinary work-related rehabilitation program. *Methods* 584 individuals on long-term sickness benefits (mean 9.3 months, SD = 3.4) were followed with official register data over a 4-year period after a rehabilitation program. 66 % were female, and mean age was 44 years (SD = 9.3). The majority had a mental (47 %) or a musculoskeletal (46 %) diagnosis. 7 % had other diagnoses. Proportional hazards regression models were used to analyze prognostic factors for the probability of being on, and the intensity of transitions between, any of the following seven states during follow-up; working, partial sick leave, full sick leave, medical rehabilitation, vocational rehabilitation, partial disability pension (DP), and full DP. *Results* In a fully adjusted model; women, those with diagnoses other than mental and musculoskeletal, blue-collar workers, and those with previous long-term sick leave, had a lower probability for being at work and a higher probability for full DP during follow-up. DP was also associated with high age. Mental diagnoses gave higher probability for being on full sick leave, but not for transitions to full sick leave. Regression models based on transition intensities showed that risk factors for entering a given state (work or receiving sickness benefits) were slightly different from risk factors for leaving the same state. *Conclusions* The probabilities for working and for receiving sickness benefits and DP were dependent on gender, diagnoses, type of work and previous history of sick leave, as expected. The use of novel statistical methods to analyze factors predicting transition intensities have improved our understanding of how the processes to and from work, and to and from sickness benefits may differ between groups. Further research is required to understand more about differences in prognosis for return to work after intensive work-related rehabilitation efforts.

## Introduction

Several specialized occupational and vocational rehabilitation programs are offered to individuals on long-term sickness benefits. Knowledge about prognostic factors for work resumption after rehabilitation is still limited, and we do not know which patients will benefit most from comprehensive work-related rehabilitation efforts [1]. Additionally, there is a lack of agreement regarding when and how work resumption should be measured [2, 3], and little is known about the long-term work outcomes after work-related rehabilitation [4, 5]. The present study was a 4-year follow-up of employees on long-term sickness benefits who had participated in an inpatient interdisciplinary work-related rehabilitation program in Norway.

Medical diagnoses related to musculoskeletal complaints and mild or moderate mental health problems are the most frequent diagnoses for long-term sick leave and disability pension (DP) in Norway [6–8], and in other industrialized countries [9, 10]. This is a heterogeneous group of patients, often with no or few objective medical explanations and with a high rate of co-morbidity with other health complaints [11–15]. They are also the main target for work-related rehabilitation.

Socio-demographic factors, work-related factors and factors related to the medical condition are the three dominating types of prognostic factors for long-term sickness benefits and return to work (RTW). Norwegian women have a higher rate of sick leave and DP than men, and still little is known on this gender divide [16, 17]. Female gender [1, 4, 18] and higher age [1, 19, 20] predict lack of RTW. Length of sick leave before rehabilitation [1] and sick leave in itself, are considered to be important risk factors for delayed RTW [19], and for future DP [21]. Both psychosocial and physical work factors predict long-term sick leave [22]. Unskilled [5, 23] and manual work, lack of job control, interpersonal relations and emotional demands are among these work factors, but differ between gender, age and socio-economic position [22]. There are some inconsistencies in how the diagnosis affects the prognosis for RTW and DP after sickness absence, and diverse diagnoses seem to affect men’s and women’s prognosis in different ways [8, 24]. In a 1-year follow-up after work-related rehabilitation, we did not find any associations between the sick leave diagnoses (musculoskeletal, mental or unspecified diagnosis) and RTW [13]. Work ability and impairment from medical diagnoses will in general be related to the perception, attribution and expectations of the individual, and are in part determined by earlier experience and learning [25]. The degree of co-morbidity may also influence the prognosis for recovery after sickness absence, and it is a risk factor for long-term incapacity for work [26].

RTW is a complex process [27], where the individual over time may have multiple and recurrent transitions between RTW and various sickness benefits [3, 28]. Recently, more emphasize has been given to the individuals mobility between different social security benefits during follow up, and how this may affect work resumption [21]. However we do not know if the variation in mobility between different benefits and work is related to specific socio-demographic factors [21], and especially gender differences in such transitions is of interest [16, 29].

The Norwegian sickness compensation system represents a generous welfare model intended to secure the income of individuals with temporary or permanent reduced function due to a disease. If you have been in paid work the last 4 weeks before the sickness incident you are entitled to sickness benefits from The Labour and Welfare Administration. An employee cannot be discharged due to sick leave; these legislations are especially strict during the first 12 months. To be entitled sickness compensation the incapacity for work must be caused by reduced functional ability due to a disease or an injury. In Norway, the general practitioner issue about 79 % of all long-term sick leaves [30], and a medical diagnosis is required on the sickness certificate. The international classification of primary care (ICPC) is the main diagnostic system used within general practice and primary care, and within The Labour and Welfare Administration. The employee receives 100 % compensation during the first year. After up to 1 year on sick leave benefit, the sick listed may be entitled a work assessment allowance. If medical or vocational rehabilitation efforts have no intended effect, the individual may be granted a DP, however partial sickness benefits are actively recommended by the authorities.

Previously, in a 4-year follow-up of patients on long-term sick leave, we used multi-state models, synthesizing the transition intensities between the different categories for sickness benefits and RTW a patient could be in after work-related rehabilitation [28]. We found an increased probability for being at work, a decreased probability for being on sick leave, and an increased probability for DP. The participants had an average of 4 transitions between work and different benefits during follow-up. The aim of the current follow-up study is to further explore the probability for work resumption and for being in, and having transitions between, work and different benefits during a 4-year follow up after participation in a work-related rehabilitation program. Age, gender, diagnosis, occupation and length of sick leave before rehabilitation, are used as predictors.

## Methods

We conducted a longitudinal cohort study of individuals on long-term sick leave, who had participated in a comprehensive, interdisciplinary work-related rehabilitation program. During 2001, 615 individuals completed the rehabilitation program. At the end of the program all these patients were invited to participate in the study. 586 individuals gave informed consent to obtain data from the patient journals and registers. Socio-demographic data at baseline was obtained from patient journals and follow-up data from official registers of The Labour and Welfare Administration in Norway. Data were missing for 2 individuals, thus 584 individuals were included. Each of these individuals was followed with register data on sickness benefits for 4 years after the stay at the rehabilitation clinic. The study was approved by the Medical Ethics Committee; Region South in Norway. All principles in the Helsinki declaration were followed.

### Participants

584 individuals, 383 (66 %) women, mean age 44 years, (SD = 9.3), who had been on different long-term sickness benefits, mean length 9.3 months, [(SD = 3.4), range 0–61 months], mainly due to musculoskeletal (46 %) and mental (47 %) diagnoses before participating in the rehabilitation program, participated in the study. 7 % had other diagnoses, with diagnoses related to neurological and heart diseases as the most common (Table 1).

**Table 1:** Description of the study population on the categories utilized for the independent variables in the regression analysis, (n = 584)

	n	%
Gender		
Male	201	34
Female	383	66
Diagnoses; codes from the ICPC-2^#^		
Musculoskeletal diagnoses	**271**	46
Back pain with or without radiating pain (L02, L03, L84, L86)	152	
Neck/shoulder/arm pain (L08, L12, L83, L92, L93)	43	
Musculoskeletal pain in general (L18, L29, L81, L82, L99)	55	
Other (L11, L15, L20, L76, L88, L90, L91, L94, L97)	21	
Mental diagnoses	**275**	47
Anxiety (P01, P74)	15	
Depression (P03, P76)	130	
Neurasthenia (P78)	119	
Other (P02, P06, P24, P28, P29, P79, P86)	11	
Other diagnoses	**38**	7
Heart disease (K02, K73, K74, K75, K76, K78, K81, K87, K92, K94)	13	
Neurology (N17, N29, N71, N79, N81, N89, N94, N99)	11	
Other (A04, A87, D75, H86, R95, S91, T73, T82, T90)	14	
Occupation	n = 579*	
Blue-collar	167	29
White-collar	136	23
Health and social workers	120	21
Education and child care	91	16
Service sector	65	11
Sick leave length before work-related rehabilitation		
0–4 months	82	14
5–8 months	195	33
9–12 months	160	28
>12 months	147	25

* Information on occupation missing on 5 individuals

^#^ICPC codes up to 29 indicate symptoms/complaints; codes from 70 to 99 indicate a verified disease/disorder

The patients ended their stay at the rehabilitation clinic between January 14, and December 23, 2001. The continuous register data was obtained for all participants from departure until December 30, 2005. During the 4-year follow-up period 6 participants died, 2 received early retirement pension, and 2 participants had passed the age of 67 years and received ordinary retirement pension, at which time these observations were censored.

### Work-Related Rehabilitation

All the participants completed a 4-week inpatient rehabilitation program. The goal of the rehabilitation program was to improve level of functioning, enhance work ability, and to increase the chances of RTW. Physicians, nurses, vocational social workers, physiotherapists, and sport pedagogues constituted interdisciplinary rehabilitation teams. The content of the program was mostly the same for all the participants, and included a combination of individual and group based interventions with physical activity, education, and cognitive behavioral modification. Increased self-confidence, coping, and learning were important objectives for all the activities. At the end of the rehabilitation program a treatment plan with RTW as the main goal, was developed together with the patient. This plan could include future participation from several stakeholders outside the rehabilitation setting, e.g. different health providers, the work place or the local health insurance office.

In Norway, inpatient rehabilitation programs are offered to individuals on long-term sick leave at risk of permanent disability. Before admittance to such programs other relevant medical examinations and treatments should have been tried in the occupational or primary health care. The specific rehabilitation program in this study was carried out at a national occupational rehabilitation clinic. Patients from the whole country could be admitted to this clinic based on referrals from their general practitioners, occupational health service or the social security offices. The program is part of the healthcare system in Norway, and was therefore offered free of charge.

To be admitted to the rehabilitation clinic the individual had to be motivated to participate in the program, and having an intentional goal and plan to resume work. Exclusion criteria were serious psychiatric disorders, undecided applications for DP, or insurance claims.

### Measures

#### Independent Variables

Information about age, gender, diagnosis (The international classification of primary care, ICPC; http://www.who.int/en/ or www.kith.no), occupation, and length of sick leave before rehabilitation were obtained from patient journals at the rehabilitation clinic. Age was used as a continuous variable divided with 5 so that the reported coefficient accounts for a 5 year increase in age. The other variables were categorized before the analysis, with the first category being used as reference in the analyses (Table 1).

#### Dependent Variables

Information about different sickness benefits was achieved from official registers and constituted 7 different variables: (1) full work, i.e. no registered benefits, (2) partial sick leave or partial medical rehabilitation allowance, (3) full sick leave, (4) medical rehabilitation allowance, (5) vocational rehabilitation allowance, (6) partial disability pension and (7) full disability pension.

The sick leave benefit constitutes 100 % of the wage loss, from the first day of reported sickness up to 1 year. The employer pays the first 16 days of a sick leave period, thereafter The Labour and Welfare Administration covers the disbursement. Sick leave days paid by the employer were not included in these analyses. If the employee has not returned to work after 1 year, he or she may receive a rehabilitation allowance, which constitutes approximately 66 % of the salary. To be eligible for medical rehabilitation allowance, there must be a certain probability to recover after medical treatment. Vocational rehabilitation allowance is granted for individuals that may benefit from vocational guidance to RTW, e.g. work training or professional re-education. From 2010 medical and vocational rehabilitation allowances have been combined and are labelled work assessment allowance. After proper rehabilitation efforts have been undertaken, the individual may be entitled DP if the work ability is reduced with at least 50 %, and caused by reduced functional ability due to a disease or an injury. In the Norwegian welfare system it is possible to work part-time and at the same time receive sickness compensation. Partial sick leave includes sickness benefits from 20 to 90 %, whereas for partial rehabilitation allowance and DP it is a 50 % lower limit. The sickness compensation legislations in Norway have been slightly changed after the time period for this study (2001–2005), but this is mostly on actions/measures and administrations, thus the claimant’s economic rights are principally the same.

### Statistical Methods

The official registers included separate data files on sick leave, medical rehabilitation allowance, vocational rehabilitation allowance and DP, and included information on partial benefits from 20 to 100 %. For each individual, start and end date on each benefit were registered. Being at work was defined as the time gap with no sickness benefits, since the registers do not contain exact information on whether a person is actually working or not. The disbursements to individuals on sickness benefits are however based on these registers, and are therefore judged to be complete and valid. The register files were merged together to form one complete event history file, thereafter it was combined with the socio-demographic information from the patient journals. Overlaps in the start and end date could occur for some registered benefits in the merged file due to administrative reasons, errors, or that individuals were receiving several different graded benefits. The file was therefore modified in accordance with a predefined ranking of the different benefits, (for details, see Oyeflaten et al. [28]). When combinations of partial benefits occurred in the registers we included only data from one of the benefits in the analysis at the same time; i.e. each individual could hence only be present in one state at one specific time. This was done in accordance with the predefined ranking, where DP had a higher rank than the rehabilitation allowances, and where sick leave had the lowest rank; e.g. an individual registered on partial DP and at the same time on partial sick leave, was defined as belonging to the partial DP group.

The analyses were based on two distinct different models. Regression models for the probabilities to be in either of the states were modeled using the observed indicator for each state for each third month in the follow-up. These models were performed using generalized models with a complementary log–log link function. The results from these analyses are presented as hazard rate ratios (HRR). During follow-up a specific individual could shift between work and different benefits several times. Each shift represents an event. Repeated events or observations may be synthesized as transition intensities. In this article we analyzed risk factors for the transition intensities using extended proportional hazards models (Cox-models) for repeated observation, presented as HRR. Thus three outcome variables are presented; the probability for being *on* each of the 7 states (being working or on different sickness benefits), and the transition intensity from and *to* work and the different benefits, during follow up. The probability for working or receiving one of the different benefits is a synthesis of all the transition intensities to and from all states in the model during the follow up.

Unadjusted analyses were first carried out to explore how the independent variables (age, gender, diagnosis, occupation and length of sick leave before rehabilitation) predicted (1) leaving (transition from), (2) entering (transition to) and (3) being on each of the 7 states, i.e. the dependent variables (full work, partial sick leave, full sick leave, medical rehabilitation allowance, vocational rehabilitation allowance, partial disability pension and full disability pension). Results from the unadjusted analyses are not reported. Finally, adjusted analyses were done to study how the independent variables influenced the probabilities and transition intensities adjusted for all the other independent variables.

Genders differences on the independent variables were analyzed with Pearson Chi square tests and *t* tests. The descriptive analyses were performed using the statistical packages PASW, version 18 (SPSS Inc. Released 2009, PASW Statistics for Windows, Version 18.0, SPSS Inc., Chicago). The regression analyses were done in Stata, version 12 (StataCorp. 2011, Stata Statistical Software: Release 12, StataCorp LP, College Station, TX). All *p* values <.05 were considered statistically significant.

## Results

Men were significantly younger [$\bar{x}$ 43 (SD = 10)] than women [$\bar{x}$ 45 (SD = 9)], (*p* = .016). Men had more frequently a musculoskeletal diagnosis (58 %) than women (41 %) and more often “another diagnosis” (10 %) than women (4 %), and women had more often a mental diagnosis (55 %) than men (32 %) (*p* < .001). There were significant differences in occupations between men and women (*p* < .001); men had more often blue-collar work (51 %) than women (16 %), women had more often health and social work (27 %) than men (8 %), and women had more often work within education and child care (19 %) than men (9 %). For white-collar work there were no gender differences. No gender differences were found for length of sick leave before participation in the rehabilitation program (*p* = .604).

During follow-up there was an annual increase in participants who returned to full work, from 10 % at departure from the clinic (n = 59) to 51 % after 4 years (n = 291). (For details see Oyeflaten et al. [28]). For partial sick leave there was an annual decrease from 20 % at departure (n = 114) to 3 % after 4 years (n = 17). The same tendency were found for full sick leave: 52 % received full sick leave at departure, after 1 year the numbers were 4 %, after 2 years it had increased to 8 % but after 4 years it was down to 3 % again. For both medical rehabilitation (MR) and vocational rehabilitation (VR) allowances there was a different pattern with relative small numbers the first year (MR: 11 %, n = 64, VR: 5 %, n = 31) and a peak between 2 and 3 years (around 20 % for both allowances), then after 4 years 2 % received medical rehabilitation and 15 % (n = 80) received vocational rehabilitation allowance. For partial DP there was an annual decrease from 2 % at departure to 11 % after 4 years, and for full DP the numbers increased from 0.5 % at departure to 16 % at 4 year follow-up.

**Fig. 1:**
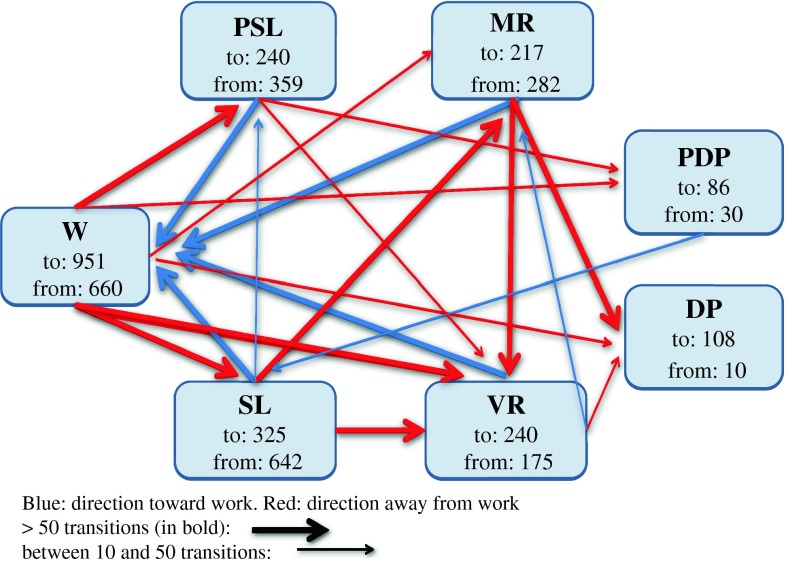
Model showing numbers and directions of transitions (above 10) to and from work and the different benefits during the 4-year follow-up. (*W* work, *PSL* partial sick leave, 100 % *SL* sick leave, *MR* medical rehabilitation, *VR* vocational rehabilitation, *PDP* partial disability pension, 100 % *DP* disability pension). n = 584

During the 4-year follow up there was a total of 2,165 transitions between work and the different sickness benefits (Fig. 1). During the total follow-up there was an average of 3.7 transitions between the different benefits and working. Median number of transitions was 3, ranging from zero to 18 transitions. (For more details see Oyeflaten et al. [28]).

### Probabilities of States and of Transition Intensities

#### Work

The probabilities for being at work during the 4-year follow-up were lower for women (HRR = 0.70, 95 % CI 0.58–0.86) than for men, and for those with “other diagnosis” (HRR = 0.62, 95 % CI 0.39–1.00) compared with musculoskeletal diagnosis (Table 2). Blue-collar workers had lower probability for being at work, compared with all other occupations; white-collar (HRR = 1.69, 95 % CI 1.29–2.22), education and child care (HRR = 1.84, 95 % CI 1.35–2.51), health and social workers (HRR = 1.63, 95 % CI 1.21–2.19), and service workers (HRR = 1.56, 95 % CI 1.11–2.18). Those with the shortest sick leave length before rehabilitation (0–4 months) showed the highest probability for being at work during follow-up, and the probability decreased with the increase in sick leave length; 5–8 months (HRR = 0.70, 95 % CI 0.56–0.89), and more than 12 months (HRR = 0.52, 95 % CI 0.39–0.70). Men, individuals working within education and child care, and those with the shortest sick leave before rehabilitation shifted more often back to work than women, blue collar workers and those with long-term sick leave, respectively (Table 2). A short sick leave length was the only predictor of transition from work.

**Table 2:** The intensity of transitions from and to 100 % work (W); Cox proportional hazards regression of relative risk (HRR), and the probability for being working (HRR) during a 4-year follow-up after work-related rehabilitation, (n = 584)

	From W	To W	In W
	HRR (CI 95 %)	HRR (CI 95 %)	HRR (CI 95 %)
Age	0.98 (0.94–1.03)	0.97 (0.92–1.03)	0.96 (0.91–1.01)
Gender			
Men	1	1	1
Female	0.97 (0.81–1.16)	**0.73** (**0.57**–**0.94**)*	**0.70** (**0.58**–**0.86**)**
Diagnoses			
Musculoskeletal	1	1	1
Mental	0.91 (0.75–1.09)	0.94 (0.74–1.19)	0.90 (0.74–1.10)
Other	0.68 (0.45–1.02)	0.63 (0.38–1.05)	**0.62** (**0.39**–**1.00**)*
Occupation			
Blue-collar	1	1	1
White-collar	0.94 (0.75–1.18)	1.19 (0.89–1.62)	**1.69** (**1.29**–**2**–**22**)**
Health and social workers	1.06 (0.83–1.37)	1.35 (0.98–1.86)	**1.63** (**1.21**–**2.19**)**
Education and child care	1.07 (0.82–1.39)	**1.51** (**1.04**–**2.18**)*	**1.84** (**1.35**–**2.51**)**
Service sector	0.75 (0.54–1.03)	0.96 (0.64–1.45)	**1.56** (**1.11**–**2.18**)*
Sick leave length			
0–4 months	1	1	1
5–8 months	**0.77** (**0.62**–**0.95**)*	**0.68** (**0.51**–**0.91**)*	**0.70** (**0.56**–**0.89**)*
9–12 months	0.91 (0.74–1.12)	0.78 (0.59–1.04)	**0.71** (**0.55**–**0.91**)*
>12 months	**0.70** (**0.54**–**0.91**)*	**0.53** (**0.38**–**0.73**)**	**0.52** (**0.39**–**0.70**)**

Fully adjusted analysis for age, gender, diagnoses, occupation, and sick leave length before work-related rehabilitation. * *p* < .05, ** *p* < .005

Bold values are statistical significant

#### Partial Sick Leave

The probability for being on partial sick leave during the 4-year follow-up increased with higher age (HRR = 1.09, 95 % CI 1.01–1.18) (Table 3). High age also indicated an increased intensity for transition both from (HRR = 1.12, 95 % CI 1.04–1.21) and to (HRR = 1.12, 95 % CI 1.03–1.21) partial sick leave during the follow-up. Those with the shortest sick leave length before rehabilitation (0–4 months) shifted more often from and to partial sick leave than those with longer sick leave. Those with the longest sick leave (>12 months) were least likely to shift to partial sick leave (HRR = 0.41, 95 % CI 0.25–0.70). However, sick leave length before rehabilitation did not give a higher probability for being on partial sick leave during follow up.

**Table 3:** The intensity of transitions from and to partial sick leave (PSL); Cox proportional hazards regression of relative risk (HRR), and the probability for being on PSL (HRR) during a 4-year follow-up after work-related rehabilitation, (n = 584)

	From PSL	To PSL	On PSL
	HRR (CI 95 %)	HRR (CI 95 %)	HRR (CI 95 %)
Age	**1.12** (**1.04**–**1.21**)*	**1.12** (**1.03**–**1.21**)*	**1.09** (**1.01**–**1.18**)*
Gender			
Men	1	1	1
Female	1.08 (0.8–1.50)	1.35 (0.92–1.96)	1.06 (0.74–1.51)
Diagnoses			
Musculoskeletal	1	1	1
Mental	0.93 (0.71–1.23)	0.96 (0.71–1.34)	0.91 (0.66–1.24)
Other	0.72 (0.61–1.42)	0.81 (0.40–1.60)	0.55 (0.24–1.29)
Occupation			
Blue-collar	1	1	1
White-collar	1.22 (0.82–1.83)	1.05 (0.64–1.70)	1.54 (0.97–2.44)
Health and social workers	1.30 (0.85–1.96)	1.23 (0.80–1.96)	1.56 (0.94–2.59)
Education and child care	1.42 (0.91–2.24)	1.23 (0.73–2.11)	1.63 (0.98–2.72)
Service sector	1.24 (0.74–2.06)	1.21 (0.71–2.12)	1.02 (0.57–1.85)
Sick leave length			
0–4 months	1	1	1
5–8 months	**0.61** (**0.41**–**0.84**)*	**0.52** (**0.34**–**0.80**)*	0.79 (0.55–1.16)
9–12 months	**0.63** (**0.46**–**0.90**)*	**0.65** (**0.45**–**0.95**)*	1.08 (0.75–1.57)
>12 months	**0.55** (**0.41**–**0.82**)*	**0.41** (**0.25**–**0.70**)**	1.28 (0.84–1.94)

Fully adjusted analysis for age, gender, diagnoses, occupation, and sick leave length before work-related rehabilitation.* *p* < .05, ** *p* < .005

Bold values are statistical significant

#### 100 % Sick Leave

The probability for being on full sick leave during the 4-year follow-up were higher for those with a mental diagnosis (HRR = 1.29, 95 % CI 1.07–1.56), compared with musculoskeletal diagnosis (Table 4). However, those with a mental diagnosis shifted less often to full sick leave (HRR = 0.72, 95 % CI 0.52–1.01) compared to those with musculoskeletal diagnosis. Blue-collar workers had higher risk for being on full sick leave than those with white-collar occupations (HRR = 0.73, 95 % CI 0.57–0.94), and health and social workers (HRR = 0.79, 95 % CI 0.62–1.00). Those with the shortest sick leave length before rehabilitation (0-4 months) had the highest risk for being on full sick leave during follow- up, and the risk for being on full sick leave decreased with the increase in sick leave length; 9–12 months (HRR = 0.56, 95 % CI 0.45–0.71), and more than 12 months (HRR = 0.16, 95 % CI 0.10–0.27). This was also the case for transition from and to full sick leave during follow-up; those with sick leave length between 0 and 4 months had a higher intensity than those on sick leave for more than 12 months.

**Table 4:** The intensity of transitions from and to 100 % sick leave (SL); Cox proportional hazards regression of relative risk (HRR), and the probability for being on 100 % SL (HRR) during a 4-year follow-up after work-related rehabilitation, (n = 584)

	From 100 % SL	To 100 % SL	On 100 % SL
	HRR (CI 95 %)	HRR (CI 95 %)	HRR (CI 95 %)
Age	0.99 (0.94–1.05)	0.99 (0.91–1.10)	1.00 (0.96–1.05)
Gender			
Men	1	1	1
Female	0.93 (0.75–1.16)	0.95 (0.71–1.32)	0.98 (0.81–1.18)
Diagnosis			
Musculoskeletal	1	1	1
Mental	0.86 (0.71–1.10)	**0.72** (**0.51**–**1.00**)*	**1.29** (**1.07**–**1.56**)*
Other	0.98 (0.63–1.51)	0.90 (0.51–1.75)	1.23 (0.89–1.69)
Occupation			
Blue-collar	1	1	1
White-collar	0.84 (0.63–1.12)	0.80 (0.53–1.22)	**0.73** (**0.57**–**0.94**)*
Health and social workers	0.93 (0.70–1.23)	0.97 (0.63–1.50)	**0.79** (**0.62**–**1.00**)*
Education and child care	0.97 (0.71–1.33)	0.97 (0.63–1.50)	0.90 (0.70–1.16)
Service sector	**0.74** (**0.55**–**0.99**)*	0.62 (0.36–1.11)	0.78 (0.59–1.03)
Sick leave length			
0–4 months	1	1	1
5–8 months	1.03 (0.81–1.31)	0.82 (0.55–1.23)	1.02 (0.83–1.26)
9–12 months	1.20 (0.91–1.50)	1.01 (0.70–1.50)	**0.56** (**0.4**5–**0.71**)**
>12 months	**0.70** (**0.24**–**0.61**)**	**0.60** (**0.35**–**0.99**)*	**0.16** (**0.10**–**0.27**)**

Fully adjusted analysis for age, gender, diagnoses, occupation, and sick leave length before work-related rehabilitation. * *p* < .05, ** *p* < .005

Bold values are statistical significant

#### Medical Rehabilitation Allowance

The probability for being on medical rehabilitation during the 4-year follow-up was higher for blue-collar workers than for education and child care workers (HRR = 0.57, 95 % CI 0.35–0.93), (Table 5). This was also the case for transition from and to medical rehabilitation. Sick leave length gave the highest probability for being on medical rehabilitation, and increased with duration; sick leave more than 12 months (HRR = 4.42, 95 % CI 2.60–7.54), but also for transition from and to medical rehabilitation.

**Table 5:** The intensity of transitions from and to medical rehabilitation (MR); Cox proportional hazards regression of relative risk (HRR), and the probability for being on MR (HRR) during a 4-year follow-up after work-related rehabilitation, (n = 584)

	From MR	To MR	On MR
	HRR (CI 95 %)	HRR (CI 95 %)	HRR (CI 95 %)
Age	0.93 (0.87–0.98)*	0.93 (0.91–1.01)	0.99 (0.92–1.08)
Gender			
Men	1	1	1
Female	1.10 (0.81–1.43)	1.02 (0.72–1.43)	1.37 (0.97–1.93)
Diagnoses			
Musculoskeletal	1	1	1
Mental	1.15 (0.88–1.50)	1.20 (0.86–1.66)	1.12 (0.81–1.53)
Other	1.20 (0.71–1.98)	1.22 (0.65–2.31)	1.04 (0.55–1.96)
Occupation			
Blue-collar	1	1	1
White-collar	0.85 (0.61–1.21)	0.82 (0.54–1.26)	0.85 (0.57–1.26)
Health and social workers	0.87 (0.60–1.30)	0.93 (0.60–1.51)	0.69 (0.45–1.08)
Education and child care	**0.60** (**0.40**–**0.92**)*	**0.60** (**0.36**–**0.98**)*	**0.57** (**0.35**–**0.93**)*
Service sector	0.75 (0.52–1.10)	0.81 (0.50–1.32)	0.86 (0.54–1.37)
Sick leave length			
0–4 months	1	1	1
5–8 months	**1.66** (**1.11**–**2.60**)*	**1.81** (**1.20**–**2.82**)*	**2.42** (**1.44**–**4.09**)**
9–12 months	**1.94** (**1.30**–**2.97**)**	**2.10** (**1.31**–**3.25**)**	**3.51** (**2.09**–**5.90**)**
>12 months	**2.06** (**1.33**–**3.21**)**	1.04 (0.61–1.83)	**4.42** (**2.60**–**7.54**)**

Fully adjusted analysis for age, gender, diagnoses, occupation, and sick leave length before work-related rehabilitation. * *p* < .05, ** *p* < .005

Bold values are statistical significant

#### Vocational Rehabilitation Allowance

The probability for being on vocational rehabilitation during the 4-year follow-up decreased with higher age (HRR = 0.76, 95 % CI 0.70–0.83) (Table 6). This was also the case for transitions from (HRR = 0.84, 95 % CI 0.81–0.91) and to (HRR = 0.84, 95 % CI 0.81–0.91) vocational rehabilitation. Blue-collar workers had a higher risk for being on vocational rehabilitation than white-collar workers (HRR = 0.58, 95 % CI 0.35–0.94). Also, blue-collar workers had a higher intensity to shift from and to vocational rehabilitation than all other occupations. Sick leave length gave the highest probability for being on vocational rehabilitation, and increased with duration; sick leave more than 12 months (HRR = 3.27, 95 % CI 1.79–6.00), but also for transition from and to medical rehabilitation.

**Table 6:** The intensity of transitions from and to vocational rehabilitation (VR); Cox proportional hazards regression of relative risk (HRR), and the probability for being on VR (HRR) during a 4-year follow-up after work-related rehabilitation, (n = 584)

	From VR	To VR	On VR
	HRR (CI 95 %)	HRR (CI 95 %)	HRR (CI 95 %)
Age	**0.84** (**0.81**–**0.91**)**	**0.84** (**0.81**–**0.91**)**	**0.76** (**0.70**–**0.83**)**
Gender			
Men	1	1	1
Female	1.05 (0.80–1.41)	1.13 (0.83–1.55)	1.09 (0.77–1.54)
Diagnoses			
Musculoskeletal	1	1	1
Mental	1.10 (0.81–1.43)	0.97 (0.71–1.33)	0.95 (0.67–1.36)
Other	0.72 (0.41–1.40)	0.70 (0.35–1.32)	0.84 (0.43–1.66)
Occupation			
Blue-collar	1	1	1
White-collar	**0.45** (**0.30**–**0.71**)**	**0.43** (**0.29**–**0.64**)**	**0.58** (**0.35**–**0.94**)*
Health and social workers	**0.60** (**0.41**–**0.85**)*	**0.50** (**0.31**–**0.71**)**	0.77 (0.48–1.22)
Education and child care	**0.53** (**0.34**–**0.84**)*	**0.51** (**0.29**–**0.74**)**	0.63 (0.36–1.10)
Service sector	**0.70** (**0.45**–**1.01**)*	0.67 (0.44–1.03)	0.82 (0.51–1.34)
Sick leave length			
0–4 months	1	1	1
5–8 months	**2.31** (**1.43**–**3.73**)**	**2.33** (**1.51**–**3.62**)**	**2.21** (**1.24**–**3.96**)*
9–12 months	**1.98** (**1.21**–**3.24**)*	**2.06** (**1.31**–**3.31**)**	**2.20** (**1.19**–**4.06**)*
>12 months	**2.61** (**1.60**–**4.30**)**	**1.75** (**1.07**–**2.91**)*	**3.27** (**1.79**–**6.00**)**

Fully adjusted analysis for age, gender, diagnoses, occupation, and sick leave length before work-related rehabilitation. * *p* < .05, ** *p* < .005

Bold values are statistical significant

#### Partial Disability Pension

The probability for being on partial DP during the 4-year follow-up increased with higher age (HRR = 1.49, 95 % CI 1.30-1.70) (Table 7). Women had a higher probability (HRR = 1.81, 95 % CI 1.00–3.26) to be on partial DP than men. Sick leave length before rehabilitation did not give increased risk for partial DP.

**Table 7:** The intensity of transitions from and to partial disability pension (PDP); Cox proportional hazards regression of relative risk (HRR), and the probability for being on PDP (HRR) during a 4-year follow-up after work-related rehabilitation, (n = 584)

	From PDP	To PDP	On PDP
	HRR (CI 95 %)	HRR (CI 95 %)	HRR (CI 95 %)
Age	**1.40** (**1.22**–**1.61**)**	**1.41** (**1.23**–**1.62**)**	**1.49** (**1.30**–**1.70**)**
Gender			
Men	1	1	1
Female	**1.66** (**1.01**–**2.75**)*	1.45 (0.91–2.40)	1.81 (1.00–3.26)*
Diagnoses			
Musculoskeletal	1	1	1
Mental	0.70 (0.44–1.11)	0.75 (0.46–1.24)	0.82 (0.45–1.49)
Other	1.21 (0.62–2.34)	1.30 (0.60–2.71)	1.50 (0.72–3.10)
Occupation			
Blue-collar	1	1	1
White-collar	0.61 (0.34–1.10)	**0.52** (**0.30**–**0.99**)*	0.53 (0.26–1.22)
Health and social workers	0.76 (0.40–1.42)	0.91 (0.50–1.71)	0.72 (0.35–1.48)
Education and child care	0.80 (0.41–1.51)	0.64 (0.31–1.32)	0.53 (0.23–1.22)
Service sctor	0.71 (0.34–1.33)	0.81 (0.40–1.71)	0.75 (0.33–1.70)
Sick leave length			
0–4 months	1	1	1
5–8 months	0.85 (0.51–1.51)	0.63 (0.31–1.25)	0.52 (0.26–1.05)
9–12 months	1.27 (0.76–2.13)	1.41 (0.76–2.60)	1.06 (0.55–2.03)
>12 months	1.25 (0.71–2.20)	1.20 (0.60–2.31)	1.18 (0.59–2.36)

Fully adjusted analysis for age, gender, diagnoses, occupation, and sick leave length before work-related rehabilitation. * *p* < .05, ** *p* < .005

Bold values are statistical significant

### Full Disability Pension

The probability for being on full DP during the 4-year follow-up increased with higher age (HRR = 1.51, 95 % CI 1.32–1.74) (Table 8). This was also the case for transition from (HRR = 1.50, 95 % CI 1.30–1.70) and to (HRR = 1.51, 95 % CI 1.31–1.73) full DP. Women had a higher probability (HRR = 2.08, 95 % CI 1.23–3.49) to be on full DP than men. This was also the case for transition from (HRR = 1.90, 95 % CI 1.04–3.50) and to (HRR = 1.84, 95 % CI 1.04–3.25) full DP. Those with other diagnosis (HRR = 4.78, 95 % CI 2.40–9.54) had higher probability for being on full DP compared with musculoskeletal diagnosis. This was also the case for transition from (HRR = 2.25, 95 % CI 1.24–4.11) and to (HRR = 2.97, 95 % CI 1.51–5.91) full DP. Blue-collar workers had higher risk for being on full DP than education and child care workers, (HRR = 0.28, 95 % CI 0.13–0.59) and health and social workers (HRR = 0.42, 95 % CI 0.21–0.84). Sick leave length before rehabilitation gave higher probability for being on DP; sick leave more than 12 months (HRR = 3.13, 95 % CI 1.51–6.46), but also for transition from and to DP.

**Table 8:** The intensity of transitions from and to 100 % disability pension (DP); Cox proportional hazards regression of relative risk (HRR), and the probability for being on DP (HRR) during a 4-year follow-up after work-related rehabilitation, (n = 584)

	From DP	To DP	On DP
	HRR (CI 95 %)	HRR (CI 95 %)	HRR (CI 95 %)
Age	**1.50** (**1.30**–**1.70**)**	**1.51** (**1.31**–**1.73**)**	**1.51** (**1.32**–**1.74**)**
Gender			
Men	1	1	1
Female	**1.90** (**1.04**–**3.50**)*	**1.84** (**1.04**–**3.25**)*	**2.08** (**1.23**–**3.49**)*
Diagnoses			
Musculoskeletal	1	1	1
Mental	0.75 (0.50–1.20)	1.06 (0.65–1.71)	1.12 (0.69–1.81)
Other	**2.25** (**1.24**–**4.11**)*	**2.97** (**1.51**–**5.91**)**	**4.78** (**2.40**–**9.54**)**
Occupation			
Blue-collar	1	1	1
White-collar	0.80 (0.42–1.50)	0.81 (0.44–1.50)	0.68 (0.39–1.17)
Health and social workers	**0.41** (**0.21**–**0.80**)*	**0.41** (**0.20**–**0.90**)*	**0.42** (**0.21**–**0.84**)*
Education and child care	0.64 (0.32–1.30)	**0.51** (**0.24**–**0.98**)*	**0.28** (**0.13**–**0.59**)**
Service sector	0.71 (0.34–1.44)	1.13 (0.61–2.14)	0.79 (0.42–1.48)
Sick leave length			
0–4 months	1	1	1
5–8 months	**2.41** (**1.25**–**4.56**)*	1.92 (0.97–3.80)	1.92 (0.97–3.80)
9–12 months	1.63 (0.81–3.34)	1.74 (0.85–3.54)	1.27 (0.62–2.60)
>12 months	**2.61** (**1.32**–**5.10**)**	**2.61** (**1.26**–**5.41**)*	**3.13** (**1.51**–**6.46**)**

Fully adjusted analysis for age, gender, diagnoses, occupation, and sick leave length before work-related rehabilitation. * *p* < .05, ** *p* < .005

Bold values are statistical significant

All the analyses were also done stratified for gender. There were some minor non-significant differences; the effects for occupations and diagnoses differed slightly between men and women. Except for that, the stratified risk estimates followed, in general, the same pattern as in the total sample. The results from the stratified analyses are therefore not reported in any further detail.

## Discussion

### Main Results

The risk for not returning to work and for receiving DP during the 4-year follow-up were associated with blue-collar work, being female, long-term sick leave length before referral to the rehabilitation clinic, and diagnoses other than mental and musculoskeletal. Receiving partial and full DP was also associated with higher age, and those with higher age were more often on partial sick leave. Young age was strongly associated with being on vocational rehabilitation allowance. Moreover, individuals with a mental diagnosis had a higher probability for being on full sick leave, but not for transitions to full sick leave. For women, the lower probability for being at work than men, was due to a lower probability for transitions to work, whereas they had not at higher probability for leaving work than men.

### Interpretation of the Prognostic Factors

As expected, older individuals had a higher probability for DP, both full and partial. They also had a higher probability for partial sick leave. There is a well-known, and strong relationship between age and DP [29, 31]. However, we found no associations between age and full sick leave. This is contrary to others who have found that age is a strong predictor for sick leave [32]. Although many studies describe age as a significant risk factor, both for sick leave and DP, research on potential causal mechanisms are lacking [32]. Among the proposed explanations is increased morbidity due to age, exclusion of elderly from the labour market, or a more lenient granting of DP with increasing age [29]. Another explanation for the association between age and DP, may be changes in the age structure in industrial countries [10], however, these changes explain only 5 % of the increase in DP [32]. In this study, we did not find any association between young age and RTW after the rehabilitation program, but it was a higher probability for being on vocational rehabilitation allowance for those with younger age. In a Swedish study, RTW after vocational rehabilitation was found to be higher for the younger age groups, particular for those below 40 years [1]. However, vocational rehabilitation allowance in Norway may differ from vocational rehabilitation Sweden.

As expected, women had higher probability for receiving both partial and full DP, and a lower probability for working during follow-up. No gender differences were found for sick leave or for the other benefits. In Norway, and in most countries with high work participation among women, there is a higher rate of sick leave and DP among women [4, 16, 17]. Numerous theories and hypothesis have been suggested to identify the reason for this gender divide [17]. Hypothesis related to work exposure, gender specific vulnerability, health factors, socio-economic factors and the “double burden” are among the hypotheses that have been proposed [16]. Theories on gender specific patterns in the process towards DP have been suggested. However, a Norwegian study did not reveal any higher risk for women than men, in the transitions from long-term sick leave to DP [16]. For transitions between work, sickness absence, unemployment and DP, only minor gender differences are reported [33]. In the Oslo Health study, the higher rates of DP among women were attributable to self-reported health, level of mental distress, working conditions, and income [34]. This is in contrast to the population-based study in Hordaland (HUSK), where self-perceived health, work factors and family situation did not explain women’s higher likelihood of DP [17]. Thus, results seem to differ between populations and studies and there is still no consensus in how to understand the gender divide in sick leave and DP. Studies on prognostic factors for RTW after diverse work-related rehabilitation interventions, show contradictory results between genders, some with better outcomes for men [1, 35], some with better outcomes for women [23], and some with no gender differences in outcome [5, 13].

Those with a mental diagnosis at the departure from the rehabilitation clinic had a higher probability for full sick leave during follow up, compared to those with musculoskeletal diagnoses. However, the intensity for transitions to full sick leave was higher for those with musculoskeletal diagnoses. This indicates that those with mental diagnoses had longer sick leave spells than the musculoskeletal group. This is in line with studies showing longer duration of sick leave for common mental disorders [36] and longer time to RTW after onset of sick leave for this patient group [37]. We also found a strong probability for full DP among those sick listed with other diagnoses than mental and musculoskeletal. This is not in accordance with previous results from a similar population of rehabilitation patients were medical diagnosis did not predict RTW [13]. According to the literature, sickness absence is due to multifactorial causes and does not depend solely on the disease [38], hence the diagnosis per se may not reflect why so many are on long-term sick leave and why some never return to paid work. Although a medical diagnosis is essential on the sick leave certificate and a premise for receiving sickness benefits and DP, the validity of this diagnosis have been questioned, especially for more complex cases of patients with subjective health complaints [39–41]. Despite this challenge, ICPC is considered a valid diagnostic system. A possible interpretation of the strong risk estimates for DP in our study found for those with other diagnoses, may be the well-defined medical characteristics of this group, as diagnoses related to neurology and heart diseases were the most common. Our findings are supported by a recent article, which explored how the medical condition influenced acceptance or rejection of the DP application [42]. Applications with well-defined medical conditions were less often rejected than complex musculoskeletal disorders [42]. Also a register based study of long-term sick listed individuals found well-defined diseases in the nervous system, respiratory system, and circulatory system, beside mental diagnosis, to be predictors of DP in a three-year follow-up [8].

As expected, blue-collar work was a main prognostic factor for not returning to work and for receiving DP. This group is represented by manual skilled and unskilled work, and the workers have often low education and high physical workload. Unfortunately we have no information about level of education in this sample. It is yet reason to believe that our findings support the social gradient in receiving DP, which may be due to an education-based selection into the work force [43]. This is in accordance with the HUSK-study, were it was a higher risk of DP among skilled and unskilled manual workers, also after adjusting for health and other work-related factors [44]. However, this is in contrast with results after a rehabilitation program for individuals on long-term sick leave, where women working in blue-collar and service/care occupations had higher RTW at 3-years follow-up, than men [23]. Also, limited evidence has been found for an effect of physically stressful work and long-term sickness absence and DP [32]. There is limited evidence about why and how the social gradient in blue-collar occupations may affect future DP, and results seem to differ between studies and populations.

Our finding, that long sick leave length before referral to the rehabilitation clinic was a strong risk factor for not returning to work, for receiving medical and vocational rehabilitation allowances, and for DP during follow-up is in accordance with the literature [1, 5, 19, 21]. The probability for transition both from and to work during follow-up was highest for those with the shortest sick leave spells, indicating that short sick leave spells may be a risk for new sick leave spells. Those with the longest sick leave length before the rehabilitation program had a lower probability for transitions from and to full sick leave, and for being on full sick leave during the follow-up. This may be understood as an effect of the “system rules”, since it is not possible to be on sick leave benefits for more than 1 year. After 1 year the sick leave recipient will be transferred to a rehabilitation allowance, and the income decreases from 100 % compensation to 66 %.

### Partial Sick Leave and Partial Disability Pension

Partial sick leave was associated with higher age and with shorter sick leave length before rehabilitation. The probability for transitions to partial sick leave decreased with length of the sick leave. This indicates that partial sick leave is used in combination with shorter sick leave spells. For partial DP there was an association with higher age. In addition, women had higher probability for receiving partial DP, but also for transitions from partial DP, indicating that partial DP may be a transitory benefit. The detected association between partial benefits, higher age, and being a female, is in accordance with the results from a Swedish study [45]. When sick leave and DP is combined with part time work, it may be beneficial, because work is considered to be generally good for the individual’s health and well-being [46]. Partial sick leave has been put forward as a treatment method for full recovery to the workforce [45].

### Clinical Implications

The study population was a selective sample of individuals who had participated in a comprehensive work-related rehabilitation program after long-lasting sick leave. They were all at a later stage of the process of sickness absence and probably had complex problems with health, low work ability and maybe a week connection to the work place. Therefore, our findings may not be generalized to sick-listed individuals in general. Nevertheless, it is important to achieve more insight in the processes leading to RTW or to DP for this selective sample of patients.

This study was not designed to test the effect of the intervention or to conclude which patients should be selected into such programs. Still, we believe that knowledge on prognostic factors and vulnerable groups may be essential for the referrals to comprehensive rehabilitation programs, for the planning of individual treatment during the rehabilitation program and for better tailoring and coordinating of follow-up interventions after such programs. Our findings that those with a mental diagnosis had a higher probability for being on full sick leave, but not for entering sick leave, suggest that special attention should be on RTWdifficulties. Likewise, the higher risk of DP for those with other and more specific diagnoses may indicate that special attention should be on factors preventing DP. The findings should be of both national and international interest for the rehabilitation teams and the stakeholders, such as the general practitioners, the occupational physicians, or the social security officers, to better judge the risk factors for not returning to work, and to implement relevant interventions. It cannot be concluded that the rehabilitation program offered did fit less with the needs of blue collar workers, women, older participants, and those with other diseases than mental or musculoskeletal diseases. As stated in the introduction, RTW after long-term sick leave may be a complex process [27], and there may be interaction effects between different prognostic factors for RTW and sickness benefits. In addition to factors on socio-demography, work and health, also personal factors related to earlier experiences and expectations may influence the prognosis for recovery and RTW [25].

### Strengths and Limitations

To our knowledge this is the first study to explore prognostic factors for transitions between work and all possible sickness benefits during a long follow-up period after a work-related rehabilitation program. The probability for any event during follow-up is a synthesis of all transitions in and out of this event, and thus we captured the whole RTW process in the prognostic models. Access to register data and socio-demographic data from the patient journals made it possible to track the total cohort of rehabilitation patients during the whole follow-up period without any drop-outs or missing data.

The study would have benefited even more if the patient journals had information about education, since the education divide may be an explanation why those with manual work have higher risk of DP [43, 44].Workplace context may also differ between different occupations and may as such be an important barrier or a facilitator for work resumption [27]. However this study only includes information about occupation; no information on work-related factors such as psychosocial factors, work tasks, or work environment was included. Access to secondary diagnosis could also have strengthened the analysis, since it is recognized that many of the rehabilitation patients have co-morbid conditions, which is an independent risk factor for long-term incapacity for work [26]. It is also a limitation that the official registers utilized in this study did not give access to unemployment benefits. However, the number of people on unemployment benefits in Norway is very low, and this was also outside the scope of this study. Additionally the register data contains little information on whether a person is actually working or not. We defined work to be the time gap between dates of different sickness benefits in the register files. Based on our analysis, we believe this to be a correct and valid interpretation [28].

## Conclusions

Among subgroups of long-term sick-listed rehabilitation patients, there were differences in the probabilities for RTW, sickness benefits and DP after participating in a work-related rehabilitation program. Blue-collar workers, women, those with previous long-term sick leave, and those with diagnoses other than mental and musculoskeletal, had a lower probability for being at work and a higher probability for full DP during follow-up. Mental diagnoses gave higher probability for being on full sick leave, but not for transitions to sick leave The current study adds to the literature by new insight into prognostic factors for transitions to and from work and sickness benefits, and how this differ between groups. However, there are still unexplained differences in the long-term RTW prognosis between men and women, occupations, medical diagnoses and different age groups. Possible interaction effects between these predictors should be investigated further, especially on how these findings are influenced by personal factors and a social gradient in health and working life. Further research is required to understand more about why there are differences in the transitions to and from work and different sickness benefits after intensive work-related rehabilitation efforts, between long-term sick listed men and women, different occupations and diagnoses.
